# Additional data and experimental setups, for a comparative study of alloys in contact to eutectic melts for thermal storage

**DOI:** 10.1016/j.dib.2021.107446

**Published:** 2021-10-04

**Authors:** Esraa Hamdy, Johanna Nockert Olovsjö, Christine Geers

**Affiliations:** a Energy and Materials, Chalmers University of Technology, Gothenburg, Sweden; b Kanthal AB, Hallstahammar, Sweden

**Keywords:** Experimental setups, Impurities, Reaction enthalpies, Post-exposure analyses, Alloy thickness loss, Phase stability

## Abstract

Three different eutectic salt mixtures have been brought into contact with three different high temperature alloys to assess corrosion damages for next-generation CSPs. This article contains additional material to support findings and assessments reported on our main article in the Solar Energy Journal [https://doi.org/10.1016/j.solener.2021.06.069]. Five sections, A-E, provide data to ensure reproducibility and confidence in our claims in the main article. A newly designed experimental setup for high temperature exposures is described as well as impurities within used chemicals. Material thickness measurements document alloy consumption by eutectic salts. Reaction enthalpies are listed illustrating individual metal species in contact with salt species at relevant temperatures. Thermodynamic single point equilibrium calculations have extended environmentally induced Laves phase precipitation found for alloy Kanthal APMT in contact with molten chlorides.

## Specifications Table

**Table:** 

Subject	Materials Chemistry
Specific subject area	Experiments and analysis of high temperature alloys in contact with molten salts for thermal storage applications
Type of data	Table Image Graph Figure
How data were acquired	Chemical specifications by and impurity analysis by the suppliers Calibrated optical camera of the Phenom ProX table-top SEM setup to determine specimen thickness loss Software: Factsage 7.3 were used [1] Energy-dispersive X-ray spectroscopy (EDX) using a JEOL JSM-7800F Prime Software: Thermodynamic equilibrium calculation (Thermocalc Software, Database TCFE:Steels/Fe-Alloys v8.0 [2]
Data format	Raw sample thickness measurements Raw EDX measurement Input from Software Databases
Parameters for data collection	Alloy coupons were exposed at 650°C or 800°C, depending on the salt melt used and quenched after completion. All post-exposure analyses have been performed in ambient conditions using standard settings of the instruments listed for data acquisition. EDS point analyses were used as input for thermodynamical phase stability calculations of precipitates found in the alloy microstructures after exposure. Corrosion reaction enthalpies for relevant phases found by XRD and potential corrosion reactions were calculated by FactSage databases and software.
Description of data collection	This DIB manuscript contains additional information to support the findings shown in the Solar Energy article [DOI: 10.1016/j.solener.2021.06.069]. The data collection is basing on duplicates to ensure minimum reproducibility.
Data source location	Institution: Chalmers University of Technology City/Town/Region: Gothenburg Country: Sweden
Data accessibility	With the article
Related research article	E. Hamdy, J. N. Olovsjö, and C. Geers, "Perspectives on selected alloys in contact with eutectic melts for thermal storage: Nitrates, carbonates and chlorides," Solar Energy, vol. 224, pp. 1210-1221, 2021/08/01/ 2021, doi: https://doi.org/10.1016/j.solener.2021.06.069.

## Value of the Data

• Experiments in molten salts are very sensitive for chemical impurities, melt loss due to evaporation, vessels and general setup qualities. Thus, a detailed description of our experimental conditions is essential to reach the necessary degree of reproducibility and applicability. Furthermore, we offer insight into our thermodynamic assessment routine on how to qualify our observations on environmentally introduced microstructural changes in our alloys.
• This additional data collection allows metallurgists, engineers and chemists working with thermal storage utilities to understand alloy consumption and microstructural changes in a more detailed way.
• This additional data complementing our main comparative study [DOI: https://doi.org/10.1016/j.solener.2021.06.069] shall provide guidance for alloy selections for thermal storage utilities utilising molten salts.
• Another interesting thought is using specific microstructural markers to assess the durability of an alloy in future plants by, e.g., electrochemical or ultrasonic online operation analysis. This data would, in this case, help significantly with the data interpretation and risk assessment.

## 1 Data Description

The additional data provided in this article is divided into five main sections; (A) sections provide detailed descriptions of the experimental procedure followed in our main study [3]. Section A1 gives the alloys preparation recipe that has been used. Table A1 and section A2 provide the reported chemical impurities of the employed salts in their respective datasheet. The chlorides purification process is thoroughly described in section A3. Section A4 comprehensively describes our newly built experimental setup of the corrosion and corrosion tests procedure. A5 summarises how the metallic samples are treated after exposure. Whereas section A6 describe different parameters and conditions used during the characterisation analysis.

**Table A1:** Impurities concentrations and chemical compositions of each salt

Salt	Moisture	Chloride (Cl^−^)	Phosphate (PO_4_^3−^)	Silicate (as SiO_2_)	Total sulfur (as SO_4_^2-^)	Calcium (Ca^2+^)	Magnesium (Mg^2+^)	Others
NaNO_3_	detected	0.0006%	1.2 ppm	-	0.0020%	0.0008%	0.0005%	Heavy metals (e.g., Pb^2+/4+^), Fe^2+/3+^ 1 ppm for each

KNO_3_	detected	0.002%	5 ppm	-	0.003%	0.005%	0.002%	Heavy metals 5 ppm, Fe^2+/3+^ 3 ppm, IO_3_^−^ 5 ppm, NO_2_ 0.001%, Na^+^ 0.005%

Li_2_CO_3_	detected	≤0.02%	-	-	≤0.05%	0.01%		Heavy metals (e.g., Pb^2+/4+^) ≤20 ppm, Fe^2+/3+^ 3 ppm

Na_2_CO_3_	Loss on drying ≤1.0%	≤0.002%	≤0.001%	≤0.002%	≤0.005 %	≤0.005 %	≤0.0005%	Heavy metals (e.g., Pb^2+/4+^), Fe^2+/3+^, N^3-/3+/5+^, Al^3+^, K^+^

K_2_CO_3_	0.113%	KCl 0.0043%	-	-	K_2_SO_4_ 12 ppm	-	-	KOH 0.106%, Na^+^ 0.20%, Fe^2+/3+^ 0.40 ppm

KCl	detected	Chlorate & Nitrate ≤0.003 %	≤5 ppm		≤0.001%	≤0.002%	≤0.001%	Ba^2+^ ≥ 0.001%, Br^−^ ≤0.01%, I^−^ ≤0.002%, Fe^2+/3+^ ≤3 ppm, Na^+^ ≤0.005%, Heavy metals (e.g., Pb^2+/4+^) ≤5 ppm

MgCl_2_	Detected 0.97%	NaCl 36 ppm CaCl_2_ 47 ppm	-	-	-	-	-	MgO (100 ppm)

Section (B) and Table B1 show the metal thickness changes of the exposed samples with a differential clarification between sample thickness, including oxide scales and the remaining metal thickness.

**Table B1:** Change in metal thickness after being exposed to different salt melts.

	Conditions				
Sample thickness after exposure	Gas	Temperature (°C)	Total exposure time (h)	Salt Melts	Measured metal thickness change (µm)
316H	Filtered air	650	1000 h	(60 wt% NaNO_3_- 40 wt% KNO_3_)	Zero metal thickness loss
Kanthal® APMT					Zero metal thickness loss
304L	CO_2_	800	1000 h	(32.1 wt% Li_2_CO_3_ -33.4 wt% Na_2_CO_3_- 34.5 wt% K_2_CO_3_)	400 µm metal thickness loss
Kanthal® APMT					Zero metal thickness loss
304L	Ar		500 h	(65 wt% KCl- 35% wt% MgCl_2_)	10–40 µm metal thickness loss
Kanthal® APMT					10 µm metal thickness loss

Section (C) gives an overview of Gibb's reaction enthalpies for metals (Al, Cr and Fe) reacting with respective alkali nitrate and alkali carbonate melts as investigated in the main article [3]. The output raw data files generated by Factsage 7.3 databases used to calculate Gibb's reaction enthalpies in Tables C1 and C2 are provided in the supplementary section.

**Table C1:** Selected reaction energies between nitrates (650°C) and relevant alloy elements correlating with experimental observations. Databases from Factsage 7.3 were used [1].

No	Equation	∆G_923K_kJ /[mol metal]
Eq.1	Al (s) + NaNO_3_(l)→ NaAlO_2_ (s) + NO (g)	−676
Eq.2	Cr (s) + NaNO_3_(l)→ NaCrO_2_ (s) + NO (g)	−376
Eq.3	Fe (s)+ NaNO_3_(l)→ NaFeO_2_ (s) + NO (g)	−258
Eq.4	8 Al (s) + 3 NaNO_3_(l)→ 3 NaAlO_2_ (s) + Al_2_O_3_ (s) + 3 AlN (s)	−539
Eq.5	8 Cr (s) + 3 NaNO_3_(l)→ 3 NaCrO_2_ (s) + Cr_2_O_3_ (s) + 3 CrN (s)	−299
Eq.6	23 Fe (s) + 4 NaNO_3_(l)→ 4 NaFeO_2_ (s) + Fe_3_O_4_ (s) + 4 Fe_4_N (s)	−88

**Table C2:** Selected reaction energies between carbonates (800°C) and relevant alloy elements correlating with experimental observations. Databases from Factsage 7.3 were used [1].

No	Equation	∆G_1073K_kJ /[mol metal]
Eq.7	2 Fe (s) + Na_2_CO_3_ (l) + 2 CO_2_(g)→ 2 NaFeO_2_ (s) + 3 CO (g)	+22
Eq.8	2 Fe (s) + Li_2_CO_3_ (l) + 2 CO_2_ (g) → 2 LiFeO_2_ (s) + 3 CO (g)	−7
Eq.9	2 Al (s) + Li_2_CO_3_ (l) + 2 CO_2_ (g) → 2 LiAlO_2_ (s) + 3 CO (g)	−414
Eq.10	2 Al (s) + Na_2_CO_3_ (l) + 2 CO_2_(g)→ 2 NaAlO_2_ (s) + 3 CO (g)	−393
Eq.11	2 Cr (s) + Na_2_CO_3_ (l) + 2 CO_2_(g)→ 2 NaCrO_2_ (s) + 3 CO (g)	−99
Eq.12	69 Cr (s) + 23 Na_2_CO_3_ (l) + 40 CO_2_(g)→ 46 NaCrO_2_ (s) + Cr_23_C_6_ (s) + 57 CO (g)	−70

Section (D) provides thermodynamic single-point calculations confirming the presence of Laves phase precipitates observed in Kanthal® APMT samples exposed to molten MgCl_2_/KCl at 800 °C, cf the supplementary files for the raw output data calculated by Thermocalc Software, Database TCFE:Steels/Fe-Alloys v8.0.

Finally, section (E) gives a summarised technical features of alloy Kanthal® APMT.

## 2 Experimental Design, Materials and Methods

### 2.1 A1 - Alloy preparation

The procedure for sample preparation was as follows: metal coupons of initial measurements 15 × 15 × 2 mm were ground using up to 1200-grit SiC abrasive paper, followed by subsequent polishing with suspensions containing 9, 3, and 1 µm diamonds to a mirror-like finish. The polished samples underwent a three-step cleaning procedure with deionised water, acetone, and ethanol using an ultrasonic bath at room temperature. Afterwards, the coupons were dried using an air gun, then dipped into the salt mixture in alumina crucibles.

### 2.2 A2 - Salts chemical composition and impurities

Eutectic mixtures were prepared using the following salts: NaNO_3_ (Alfa Aesar 99.0%), KNO_3_ (Alfa Aesar 99.0%), Li_2_CO_3_ (VWR chemicals 99.0%), Na_2_CO_3_ (EMSURE anhydrous, 99.9%), K_2_CO_3_ (ThermoFisher Scientific 99.8%), KCl (Alfa Aesar 99.0%), MgCl_2_ (Alfa Aesar anhydrous 99.0%). Suppliers provide the impurities concentrations in the salts. Table A1 summarises the impurities measured in the salts and has been reported in the salts' chemical datasheets.

### 2.3 A3 - Chlorides’ purification process

The stepwise thermal purification process utilised in this work Ref. [main article] was based on previous studies [4], [5], [6]. The purification process conducted in this study followed these steps; (i) The chloride mixture was first dried at 100 °C for at least five hours. (ii) Afterwards, the temperature was increased to 200 °C and after a two-hour dwell time. (iii) The temperature was increased even further to 300°C and kept for another two hours. (iv) The setup was left to cool down to room temperature, then samples were dipped into the salt-containing crucible. (v) Later, the vessels are purged with Ar for 12 h and heated up to 120 °C for at least 12 h. (vi) Finally, the temperature is raised to 750 °C and kept for one hour before starting the exposure at 800 °C.

### 2.4 A4 - Corrosion tests procedure and experimental setups

In this study, two setups were employed, a horizontal silica tube furnace and the Nabertherm setup. In this section, detailed corrosion tests procedure for each setup are provided.

(i) Horizontal tube furnace

This setup was used for partial immersion corrosion tests of alloys exposed to nitrates melt. After following the cleaning procedure of the alloy sample (coupon), the coupon is dipped into an alumina crucible filled with the salts' mixture. Before exposure and to prevent contamination, samples were purged in filtered air for at least five hours. After exposure, the salt was drained from the metal coupons using a heat gun. The heating gun method was done to avoid leaching corrosion products by washing the coupons with water. However, using a heat gun was not possible for samples exposed to carbonates and chlorides because of the significantly higher melting points of carbonates and chlorides than the nitrates melt, as shown in Table 2 in our main article [3]. The crucibles were limited in volume and had to be refilled with salt every 72 h, and the samples were only partially immersed in salt. Consequently, a new setup was built to address these limitations.

(ii) Vertical vessel setup

The second setup used for complete immersion experiments was a top-loader furnace (model top 60 Nabertherm). The Nabertherm furnace was purchased and redesigned in the workshop to comply with carbonate and chloride exposures in controlled gas environments. The furnace lid holds were designed to contain two cylindrical vessels, as shown in figure A. The inner diameter for each cylinder is 80 mm with 250 mm height, and they were constructed from stainless steel 253MA.

**Fig. 1:**
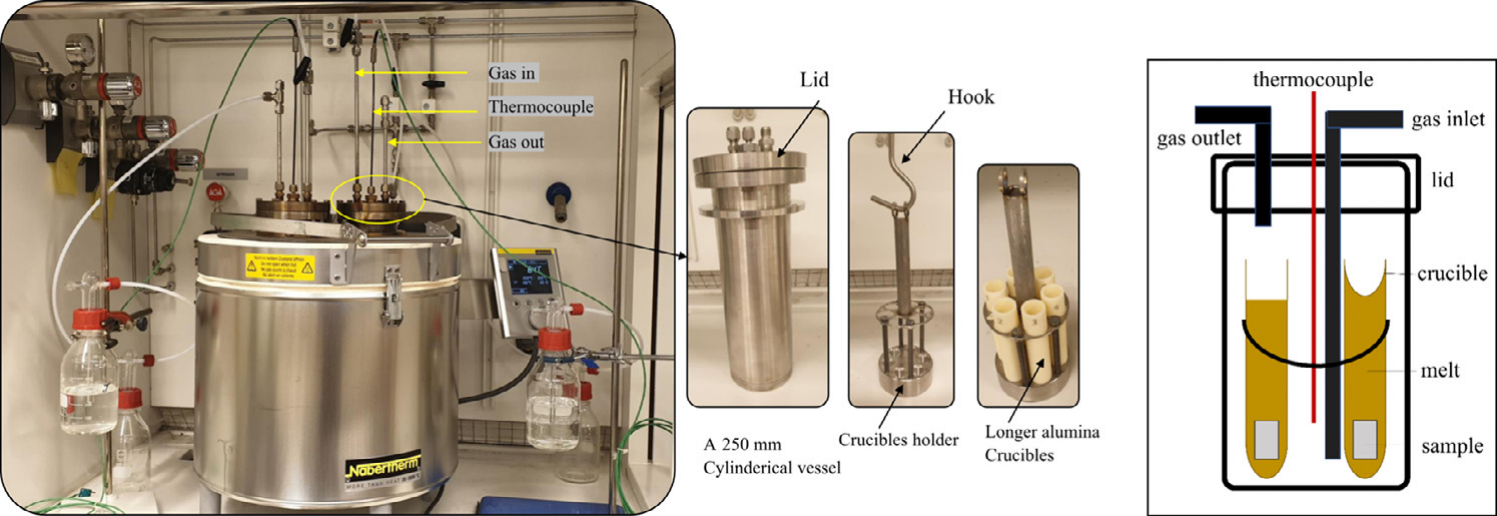
(i) New laboratory setup built for the carbonate and chloride exposures (ii) cylindrical vessel components iii) schematic diagram of the cylindrical vessel.

Aluminium-diffusion coated using a powder pack cementation process has been applied to increase both vessels' durability [7]. The vessel's lid was designed to allow gas flow in and out of the vessel and mount a thermocouple for calibration. A 75 mm diameter sample holder was machined with slots to accommodate six alumina crucibles, as shown in Fig. 1. The purpose of designing two cylindrical vessels is to duplicate the number of samples that can be tested. Each cylindrical vessel enables investigating six samples/alloys under the same conditions.

Prior to exposure, the flow rate was calibrated with a Bios Definer 220M, and the gas line was extended through the vessel lid so that the gas could flow below the crucibles. The temperature was kept above 100 °C before exposure for at least five hours to ensure the absence of water vapour in the system. The system was purged for at least 5 h and 12 h for the carbonate and chloride exposures, respectively, to avoid contamination. CO_2_ was utilised as the gas flow to suppress the decomposition of the carbonate melts. The coupons were placed vertically in alumina crucibles that had been filled with the salt mixture.

### 2.5 A5 - Sample post-exposure treatment

After exposure, alloy samples have been treated in two ways depending on the characterisation technique required. Since the vertical setup was designed to provide duplicate samples, one sample was washed with water, then weighed before and after the exposure using a SartoriusTM balance with microgram resolution. Instead of washing, the duplicate sample was left with a corrosive salt film on its surfaces after pouring off the residual melt. The procedure for the melts removal from the duplicate sample followed the upcoming steps: the temperature was lowered and maintained at 50 °C higher than the eutectic melting point for the mixture after the required exposure was completed. Holding the temperature at 50 °C higher than the eutectic melting point enabled us to pour off the salts while they were in their molten phase. Only a minor amount remained on the sample surfaces, and this salt was collected as well. The second cylindrical vessel provided a duplicate sample that was treated differently. The surface of the duplicate sample was rinsed with water to allow for mass change measurements. Each exposure was conducted twice.

According to the standard methods [8], the sample washing procedure was conducted: (i) Samples were sonicated for ten minutes at room temperature. (ii) After five minutes, sonication was interrupted. (iii) If there was salt remaining, the sample was gently brushed to remove the salts remaining, (iv) the sonication process was resumed to assure a complete dissolution of the salts. Results based on washed samples, e.g., XRD analysis and weight change values, require careful consideration. Weight change values have not been considered reliable data for corrosion evaluation, but only as an additional data point for the overall evaluation performance.

### 2.6 A6 - Characterisation techniques

Washed samples were weighed and characterised with scanning electron microscopy (SEM) and energy-dispersive X-ray spectroscopy (EDX) using a JEOL JSM-7800F Prime or Phenom ProX Desktop SEM equipped with an EDX detector. The electron beam was operated at an accelerating voltage of 15 kV, and collected EDX spectra. A Siemens D5000 powder diffractometer with grazing-incidence geometry was used for XRD surface analysis.

Unwashed samples were subjected to cross-section investigation. Cross-sections of the exposed samples were prepared by dry cutting with a low-speed diamond saw, followed by broad ion beam (BIB) milling with a Leica TIC 3X instrument. This device is equipped with three argon-ion guns for sputtering. The guns were operated at 8V, and the total sputtering time was seven hours. Before milling, the samples were sputter-coated with gold, and a thin polished silicon wafer was affixed to the surface to protect the oxide scale during milling.

### 2.7 B - Metal thickness changes

Table B1 summarises metal thickness changes to 316H, 304L, and Kanthal® APMT exposed to nitrate, carbonate, and chloride melts in this section. A calibrated optical camera of the Phenom ProX table-top SEM setup was employed to determine specimen thickness loss. It is essential to distinguish between the overall sample thickness, including oxide scales and the remaining metal thickness.

#### 2.7.1 Nitrates

Alloy 316H and Kanthal® APMT exposed to 60 wt% NaNO_3_- 40 wt% KNO_3_ have not shown any loss in metal thickness; this agrees with their high corrosion resistance to the nitrate melts as reported in the main article's chapter 3.1.[3]

#### 2.7.2 Carbonates

Despite the increase in total thickness of the 304L sample due to rapidly outward growing oxides, almost 400 µm of 304L metal thickness was lost. It is noteworthy that the remaining thickness of metallic components in 304L has been internally attacked and completely carburised, which alters the overall alloy chemistry. In comparison, Kanthal APMT thickness has not changed after being exposed to the carbonate melt.

#### 2.7.3 Chlorides

The metal thickness loss in 304L and Kanthal APMT, corroded by the 65 wt% KCl- 35% wt% MgCl_2,_ has been assigned to metallic elements leaching, and this could be observed as cavities in the bulk alloy. Unlike 304L exposed to carbonate melt, the cavities have not changed the chemistry of both alloys. Hence, the measured metal thickness for alloy 304L and Kanthal APMT included internally attacked zones comprising of MgO filled cavities. The metal thickness loss measured for 304L and Kanthal APMT was up to 10–40 µm and 10 µm, respectively.

### 2.8 C – Reaction enthalpies

Overview of Gibb's reaction enthalpies for metals (Al, Cr and Fe) reacting with sodium nitrate at 650 °C in Table C1 and an equivalent overview in Table C2 for metals reacting with carbonates in a CO_2_ gas atmosphere at 800°C. The data has been normalised to per mol metal reacting with salt, which means that, e.g., the reaction enthalpy of Eq. (4) has been divided by 8. This provides direct comparability between all reactions. Factsage 7.3 databases were used to generate the data.

**Table D1:** Thermodynamic equilibrium calculation (Thermocalc Software, Database TCFE:Steels/Fe-Alloys v8.0[2]) for the suboxide scale composition indicated in Fig. 11 (b) in the main article [DOI: 10.1016/j.solener.2021.06.069] normalised to 1 mol. (a,b) Laves phase elemental composition and (c) sublattices occupation at equilibrium at 800°C. d) Theoretical temperature dependent equilibrium phase composition plot for EDX measured composition (a) normalised to 1 mol (Thermocalc Software package and database TCFE: Steels/Fe-Alloys v8.0). (Thermocalc Software, Database TCFE:Steels/Fe-Alloys v8.0 [2])

(a) Measured input values			(b) Elemental composistion of the Laves phase C14#1 Constituents of the Laves phase		
Element	Mole Fraction	Mass Fraction	Element	Mole Fraction	Mass Fraction
Mo	0.1	0.167	Mo	0.333	0.469
Al	0.03	0.014	Fe	0.484	0.396
Cr	0.25	0.227	Cr	0.170	0.130
Si	0.024	0.012	Si	0.013	0.006
Fe	0.596	0.580	Al	1.44E-06	5.7E-07

Output: Thermocalc single point calculation at 800°C:					

BCC	68 mass% (73.3 mol%)				
Laves phase C14#1	32 mass% (26.7 mol%)	

(c) Sublattice constitution for Laves phase C14#1 (Al,Cr,Fe,Mo,Si)_2_(Al,Cr,Fe,Mo,Si)					

Sublattice 1:			Sublattice 2:		
Constituent	Site Fraction		Constituent		Site Fraction

Fe	0.725		Mo		0.998
Cr	0.254		Cr		0.002
Si	0.020		Fe		3.10E-04
Mo	3.28E-04		Al		2.12E-06
Al	1.10E-06		Si		4.54E-08

(d) Temperature dependent phase composition plotted for EDX input values from (a).					

### 2.9 D – Thermodynamic single-point analysis of Laves phase precipitates

The exposure of a FeCrAl alloy, Kanthal® APMT, to molten MgCl_2_/KCl presents with molybdenum rich precipitates in the suboxide region (Fig. 11b, [3]). EDS spot analyses on several precipitates were used to create input compositional data (Table D1a) for a Thermocalc single point equilibrium calculation [2]. The output file indicates a two-phase region consisting of 68 wt% BCC and 32 wt% Laves phase (A_2_B). The major fraction of molybdenum constitutes the B-sublattice in the Laves phase forming approximately (Fe_0.75_Cr_0.25_)_2_Mo, see Table D1b,c).

Part d) in Table D1 shows a uni-axial equilibrium calculation which extends the single point calculation by the dimension of temperature. Laves phase is thermodynamically stable up to 900 °C. Interestingly, the formation of a ternary sigma phase is possible below 800 °C, reducing the fraction of the BCC phase but not Laves phase.

### 2.10 E – Description of alloy Kanthal® APMT

Kanthal® APMT is a powder metallurgically produced alumina forming ferritic stainless steel. The alloy is available in several product forms. Kanthal APMT has excellent oxidation properties in air and good stability at high temperatures. At lower temperatures (i.e., below 1000 °C), it is microstructurally stable. Some product might experience secondary recrystallisation at temperatures above 1000 °C. The high resistance of the alloy to oxidation and carburisation makes it useful in many demanding environments at elevated temperatures [9], [10], [11]. Kanthal APMT might not be cost-competitive with other conventional stainless steels, as its cost is roughly 20 times more expensive per kg than 304L and 316 alloys. Nevertheless, Kanthal APMT is considered a promising candidate for the next generation of CSP plants due to the following. (i) its high corrosion resistance in different molten salts compared to 304L and 316H alloys. (ii) the necessity to deviate from stainless steels due to unacceptable material loss and catastrophic failure risks.

## Ethics Statement

Hereby, the authors assure that the manuscript adheres to Ethics in publishing standards.

## CRediT Author Statement

**Esraa Hamdy:** Conducting exposure experiments, improving setup design, sample analyses, main author of this article; **Johanna Nockert Olovsjö:** Sample material advisor and main project partner for supplying Kanthal® APMT, prototype vessel production, co-authoring this article; **Christine Geers:** Conducting exposure experiments, improving setup design, sample analyses, thermodynamic calculations, co-authoring this article.

## Declaration of Competing Interest

The authors declare that they have no known competing financial interests or personal relationships that could have appeared to influence the work reported in this paper.

## Appendix Supplementary materials

Image, application 1

Image, application 2

Image, application 3
