# Supplementary figures and images for: Additional data and experimental setups, for a comparative study of alloys in contact to eutectic melts for thermal storage
Source: Data Brief. 2021 Oct 4;38:107446. doi: 10.1016/j.dib.2021.107446 (PMC8517162; doi:10.1016/j.dib.2021.107446)

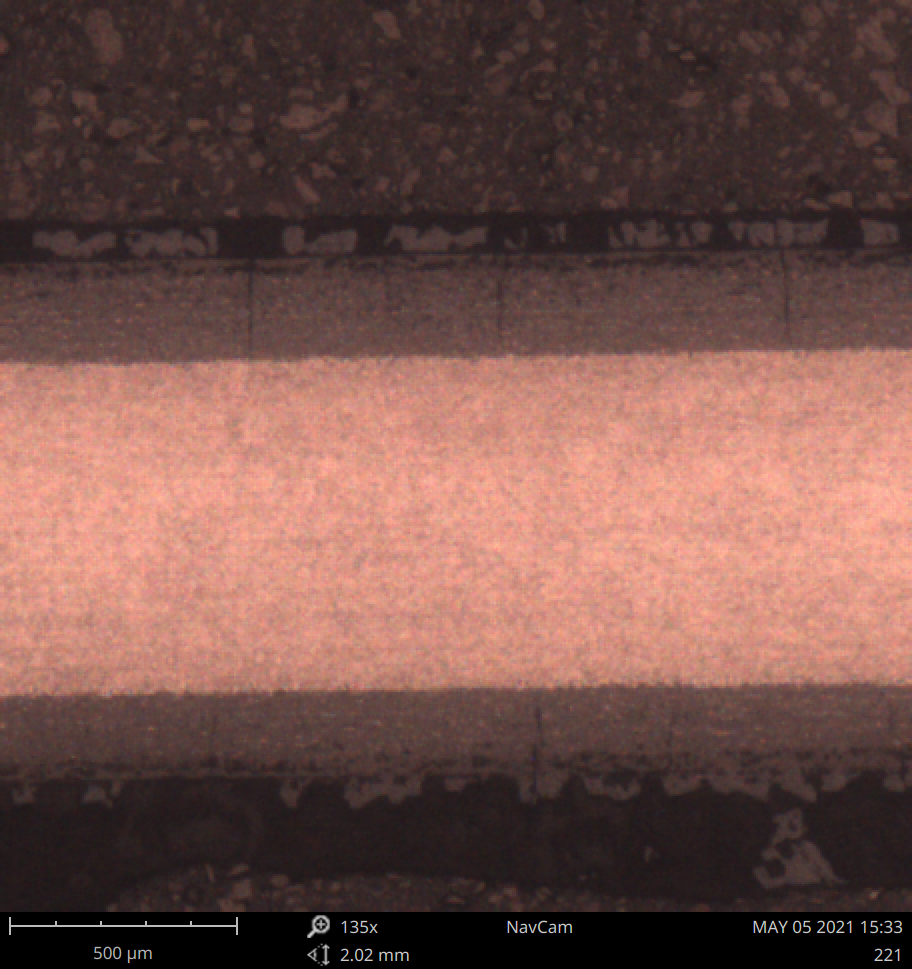

Supplement: Supplementary file 1 [file mmc1.zip › Raw files,figures/304L, carbonate, high mag2210003.tiff]

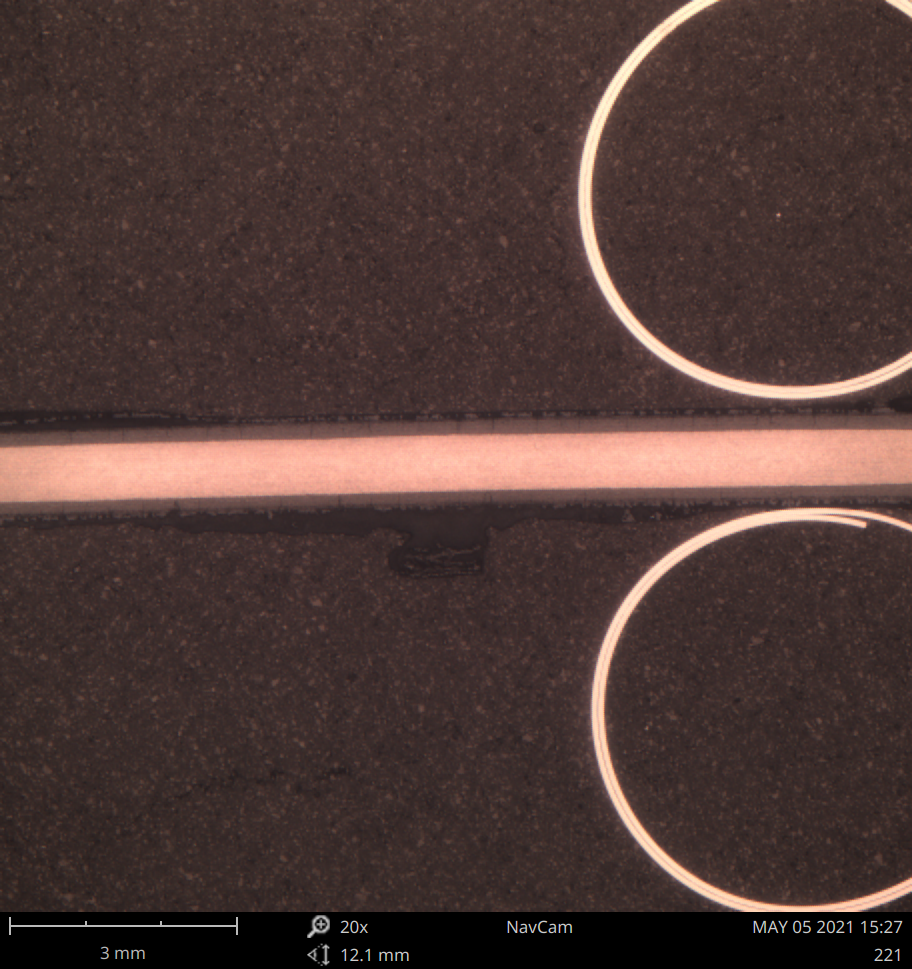

Supplement: Supplementary file 1 [file mmc1.zip › Raw files,figures/304L, carbonate, low mag 2210001.tiff]

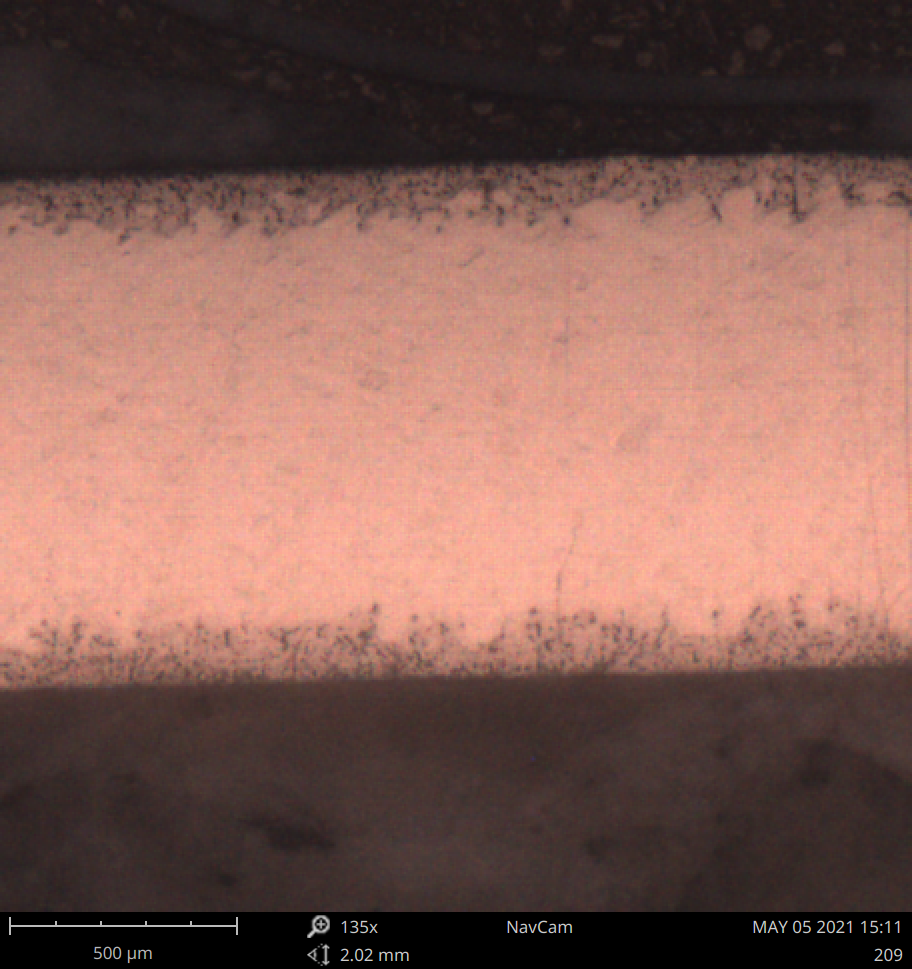

Supplement: Supplementary file 1 [file mmc1.zip › Raw files,figures/304L, chlorides, high mag 2090003.tiff]

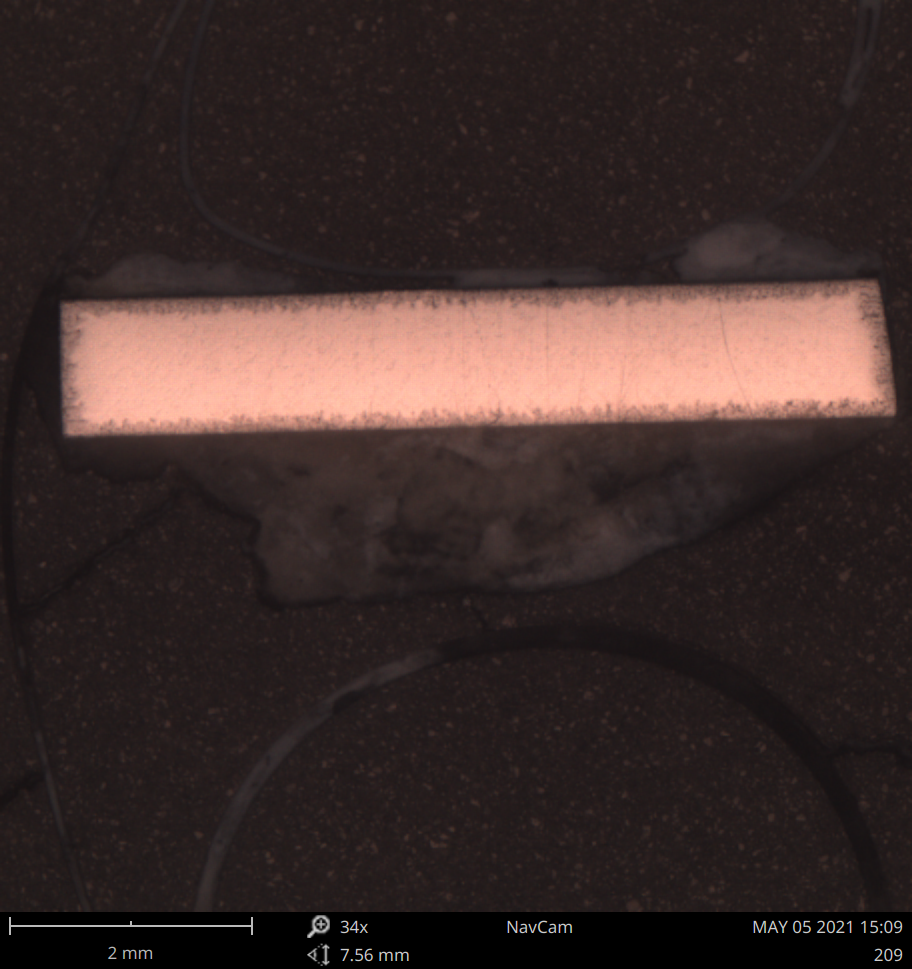

Supplement: Supplementary file 1 [file mmc1.zip › Raw files,figures/304L, chlorides, low mag 2090002.tiff]

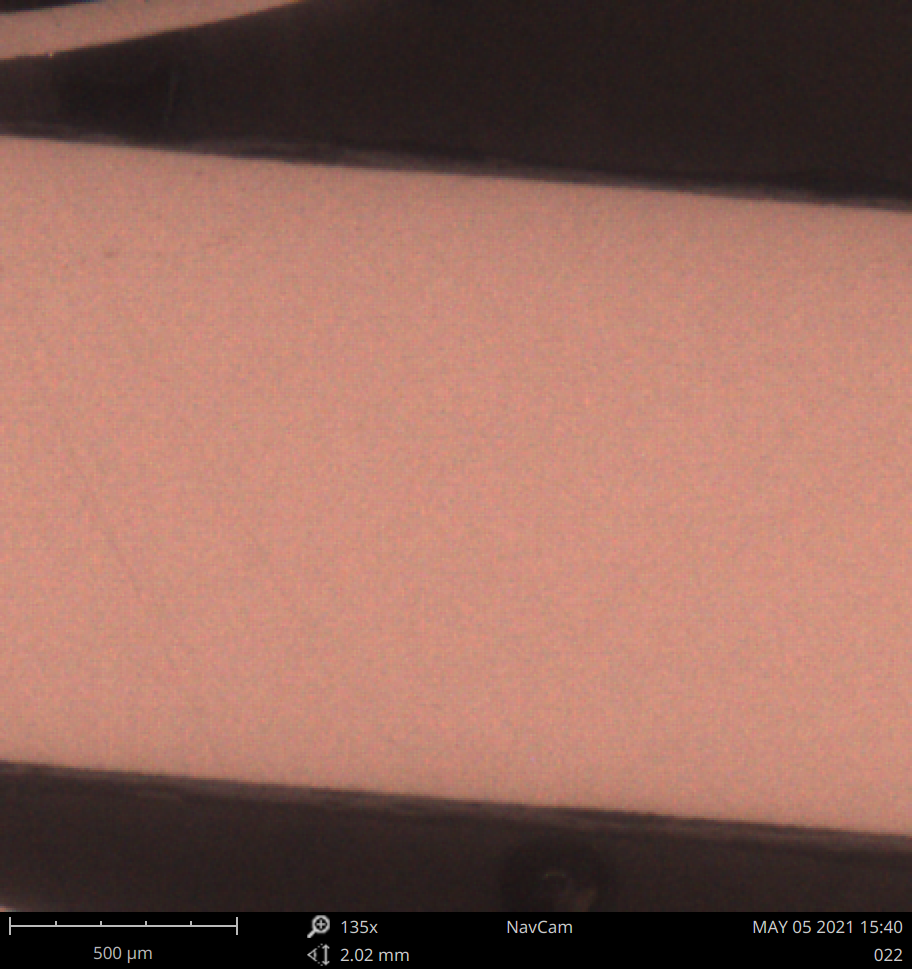

Supplement: Supplementary file 1 [file mmc1.zip › Raw files,figures/316H, nitrate, high mag 0220002.tiff]

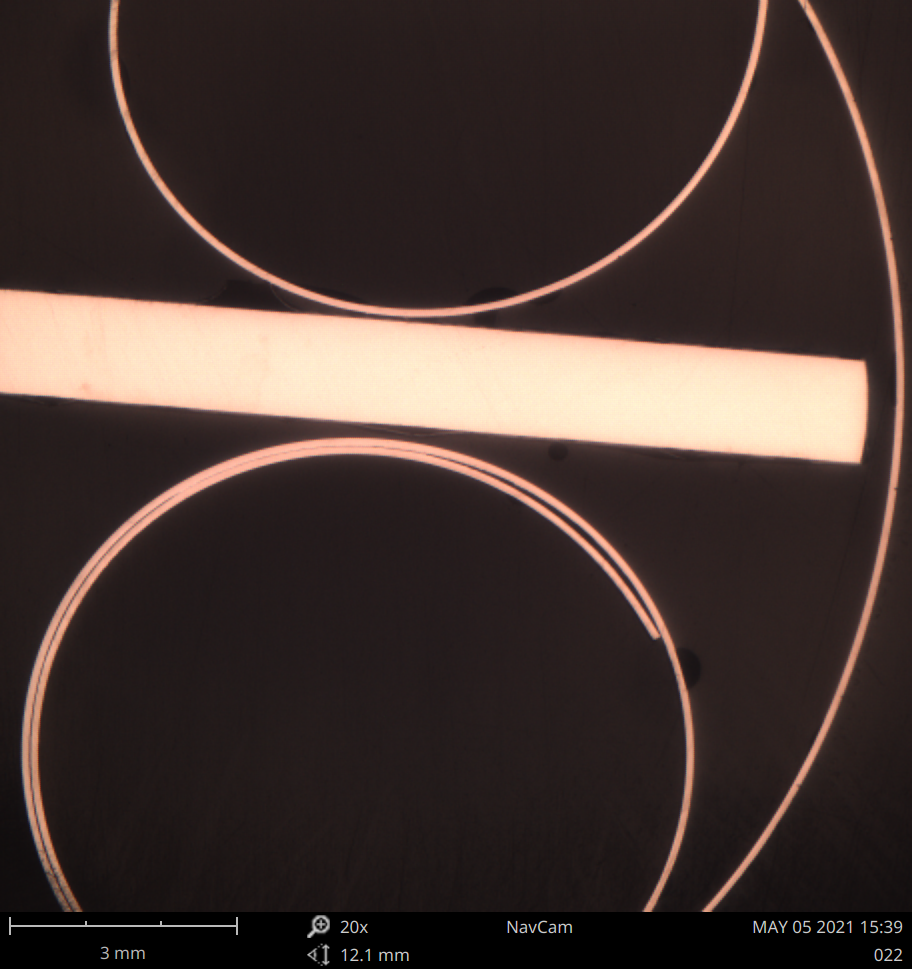

Supplement: Supplementary file 1 [file mmc1.zip › Raw files,figures/316H, nitrate,low mag 0220001.tiff]

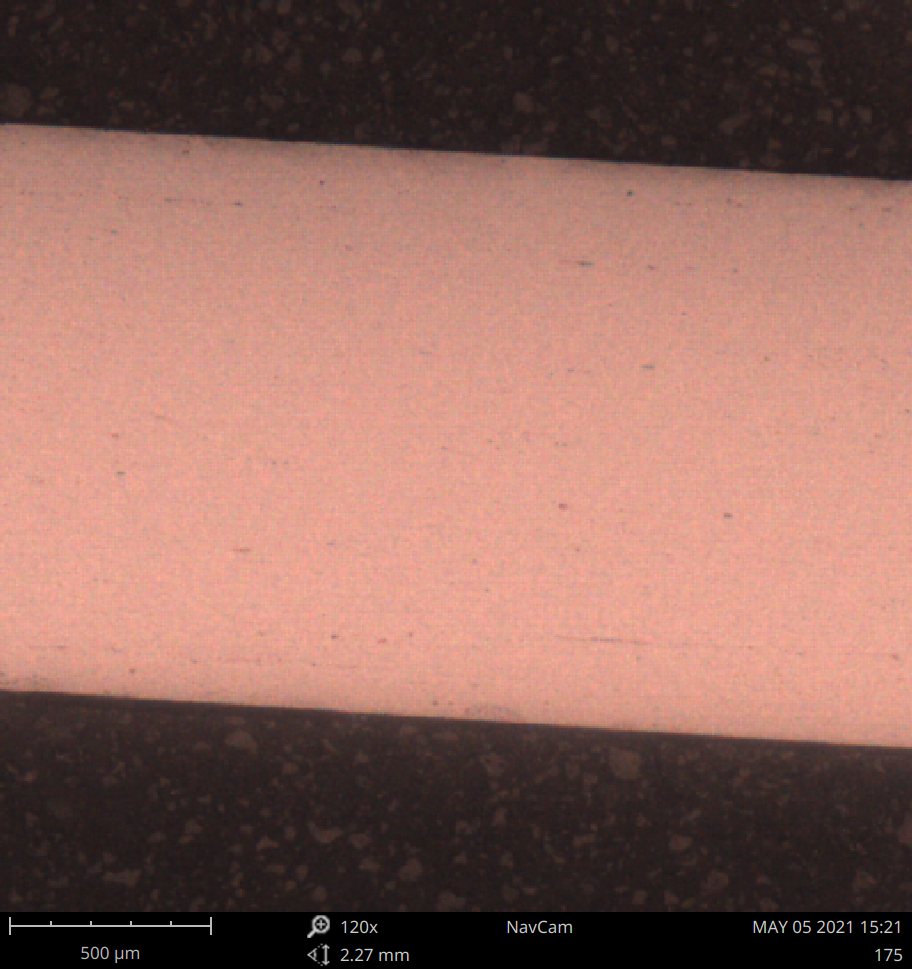

Supplement: Supplementary file 1 [file mmc1.zip › Raw files,figures/APMT, carbonate, high mag 1750002.tiff]

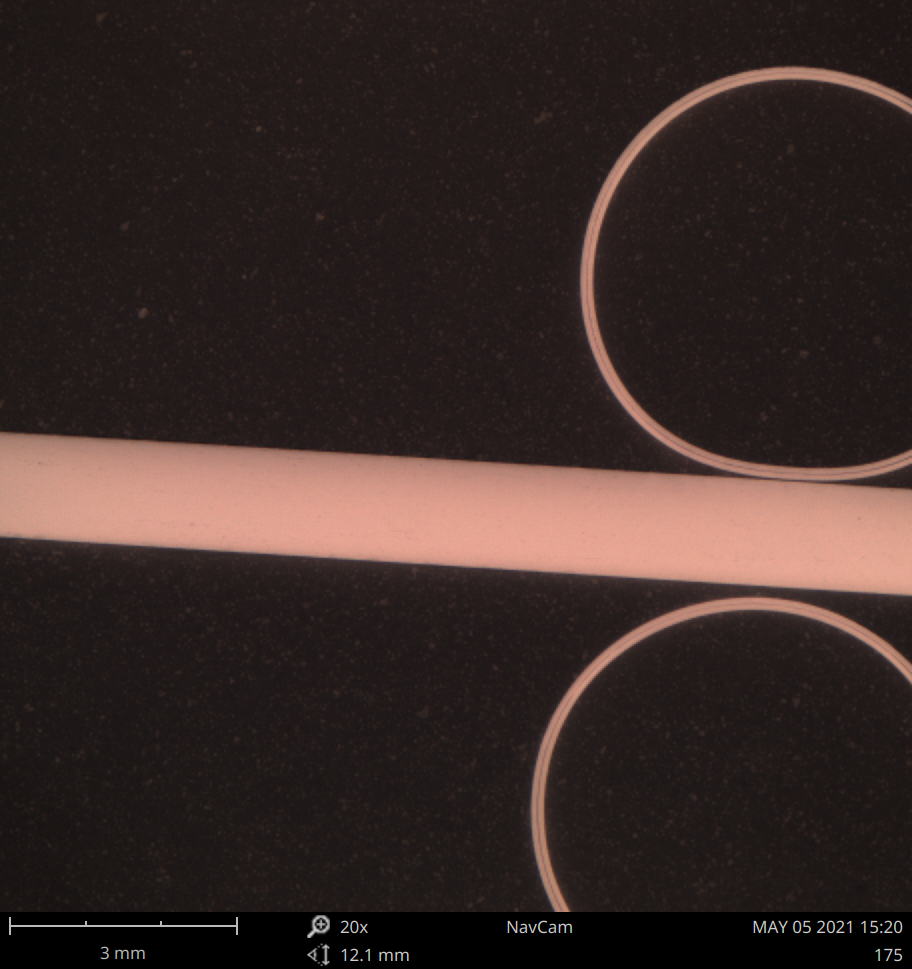

Supplement: Supplementary file 1 [file mmc1.zip › Raw files,figures/APMT, carbonate, low mag1750001.tiff]

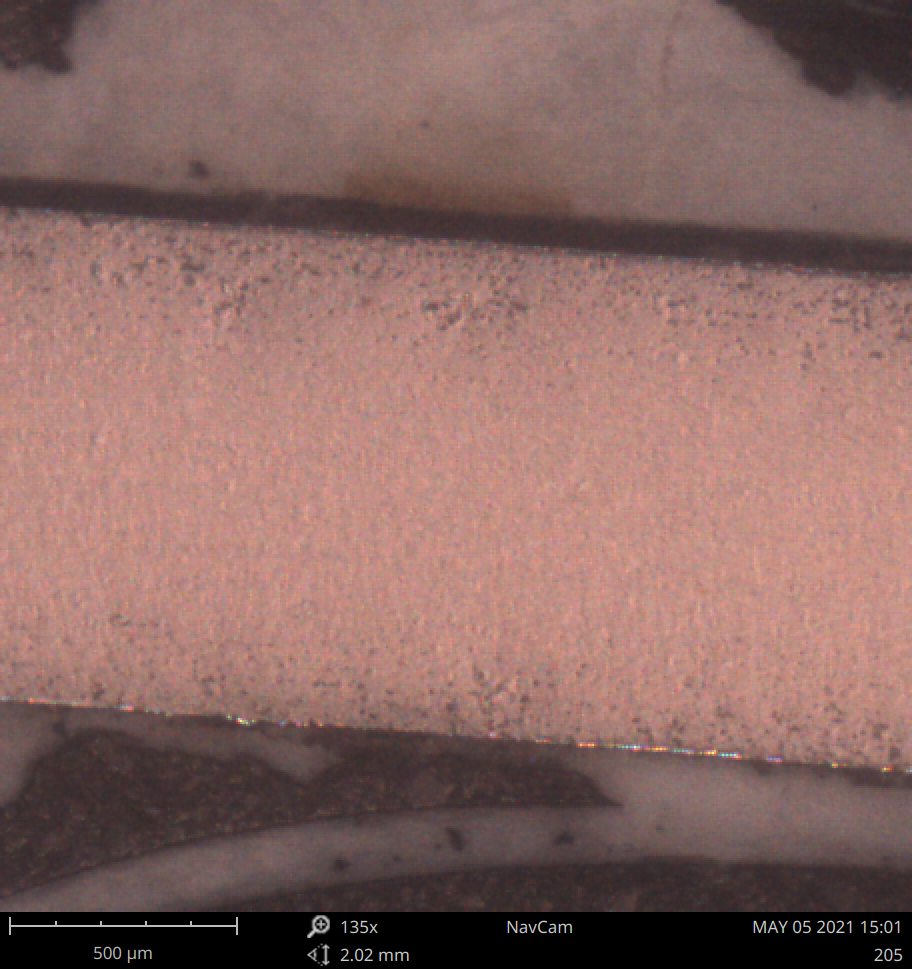

Supplement: Supplementary file 1 [file mmc1.zip › Raw files,figures/APMT, chlorides, high mag 2050005.tiff]

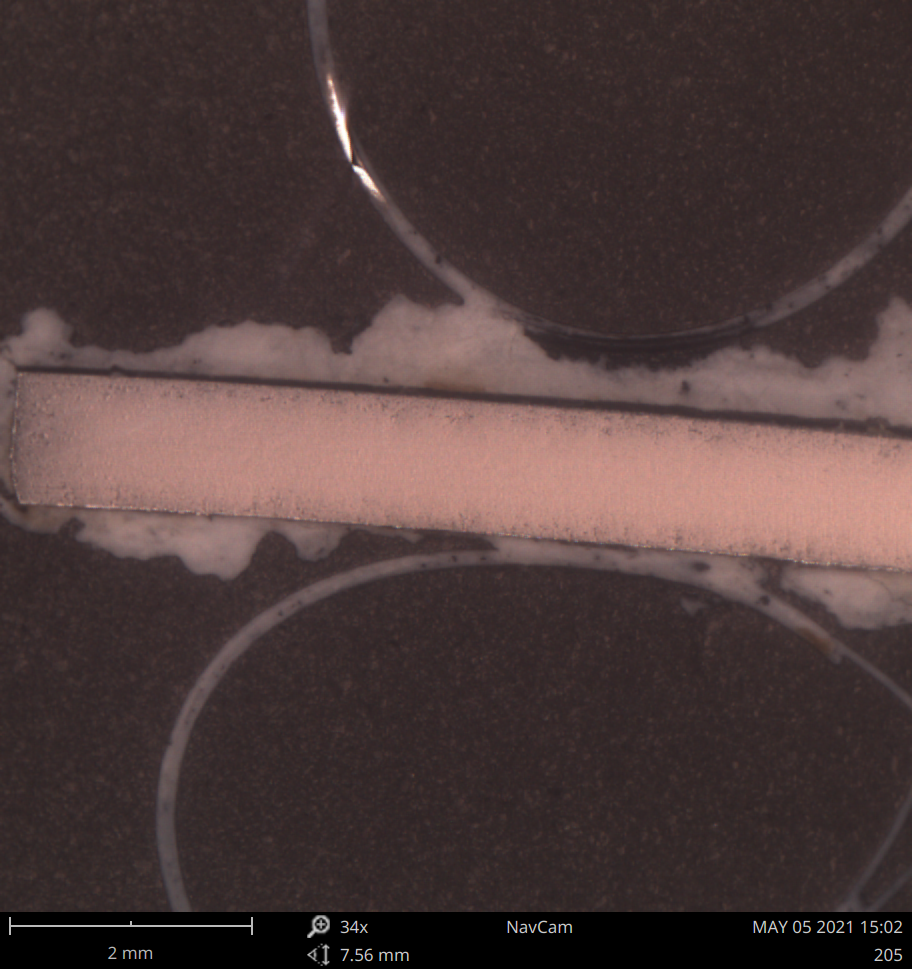

Supplement: Supplementary file 1 [file mmc1.zip › Raw files,figures/APMT, chlorides, low mag 2050006.tiff]

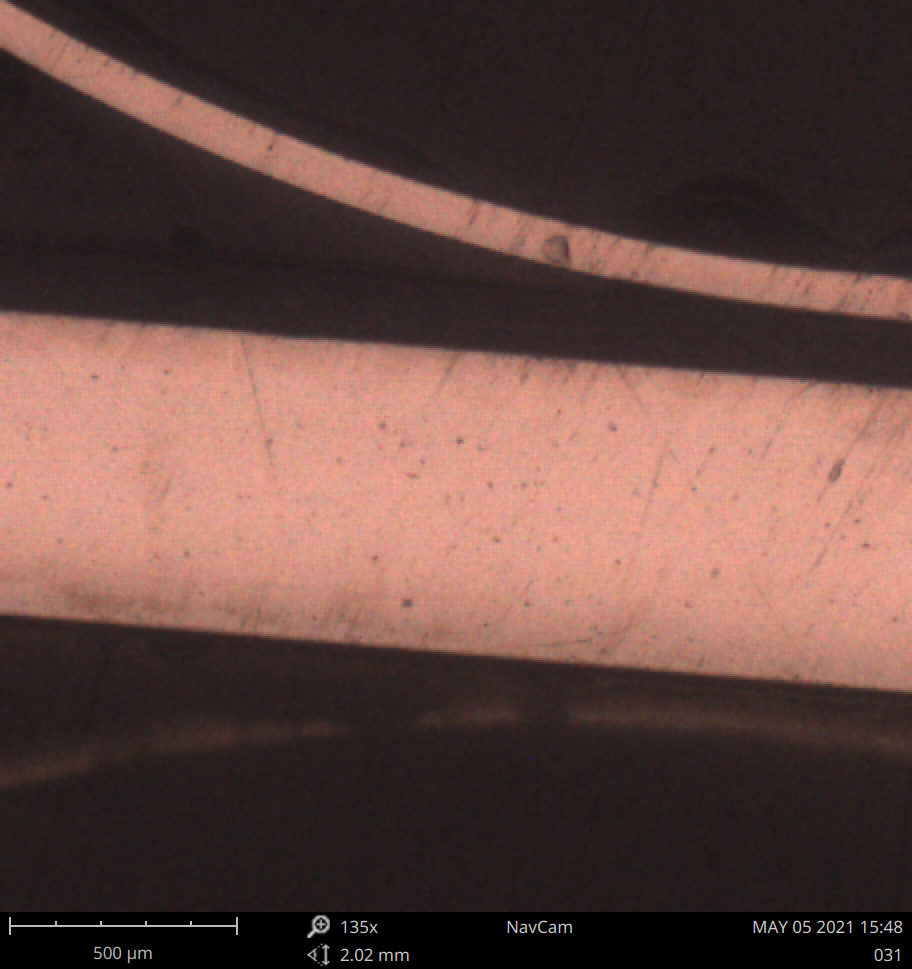

Supplement: Supplementary file 1 [file mmc1.zip › Raw files,figures/APMT, nitrate, high mag 0310002.tiff]

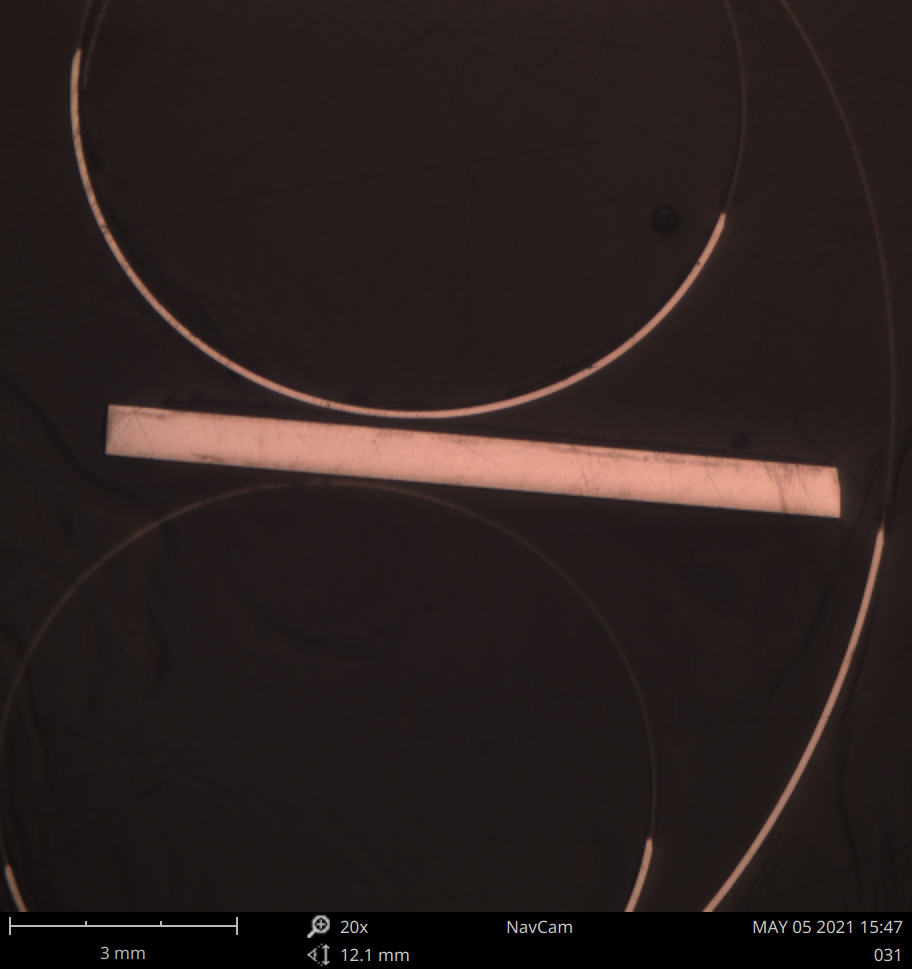

Supplement: Supplementary file 1 [file mmc1.zip › Raw files,figures/APMT, nitrate, low mag 0310001.tiff]

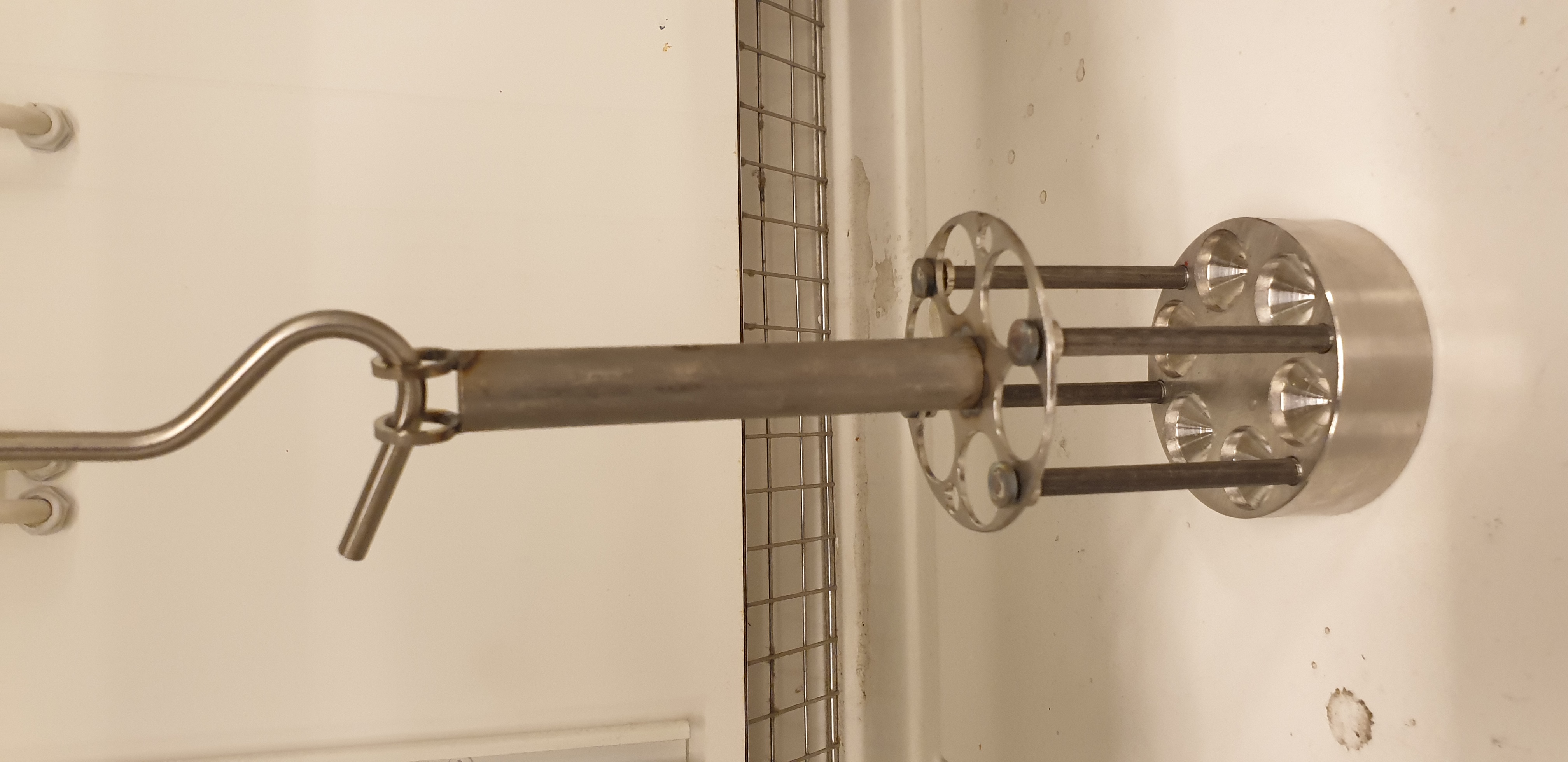

Supplement: Supplementary file 1 [file mmc1.zip › Raw files,figures/crucible holder.jpg]

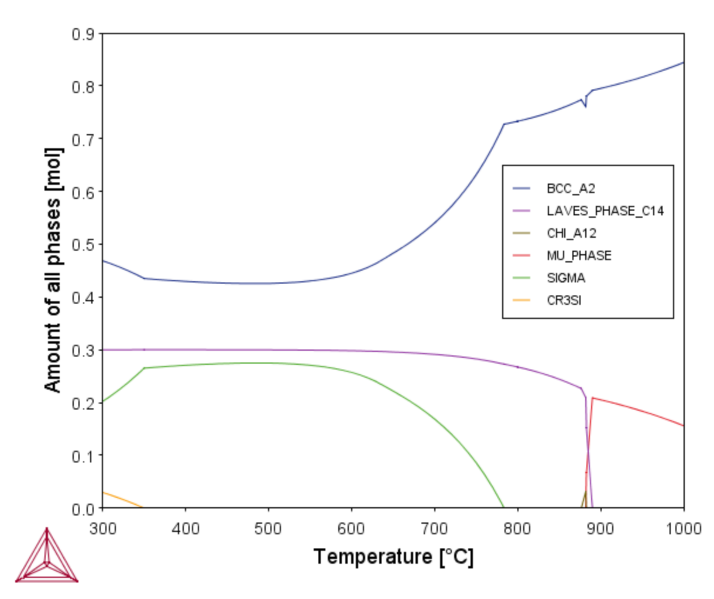

Supplement: Supplementary file 1 [file mmc1.zip › Raw files,figures/Laves phase.tiff]

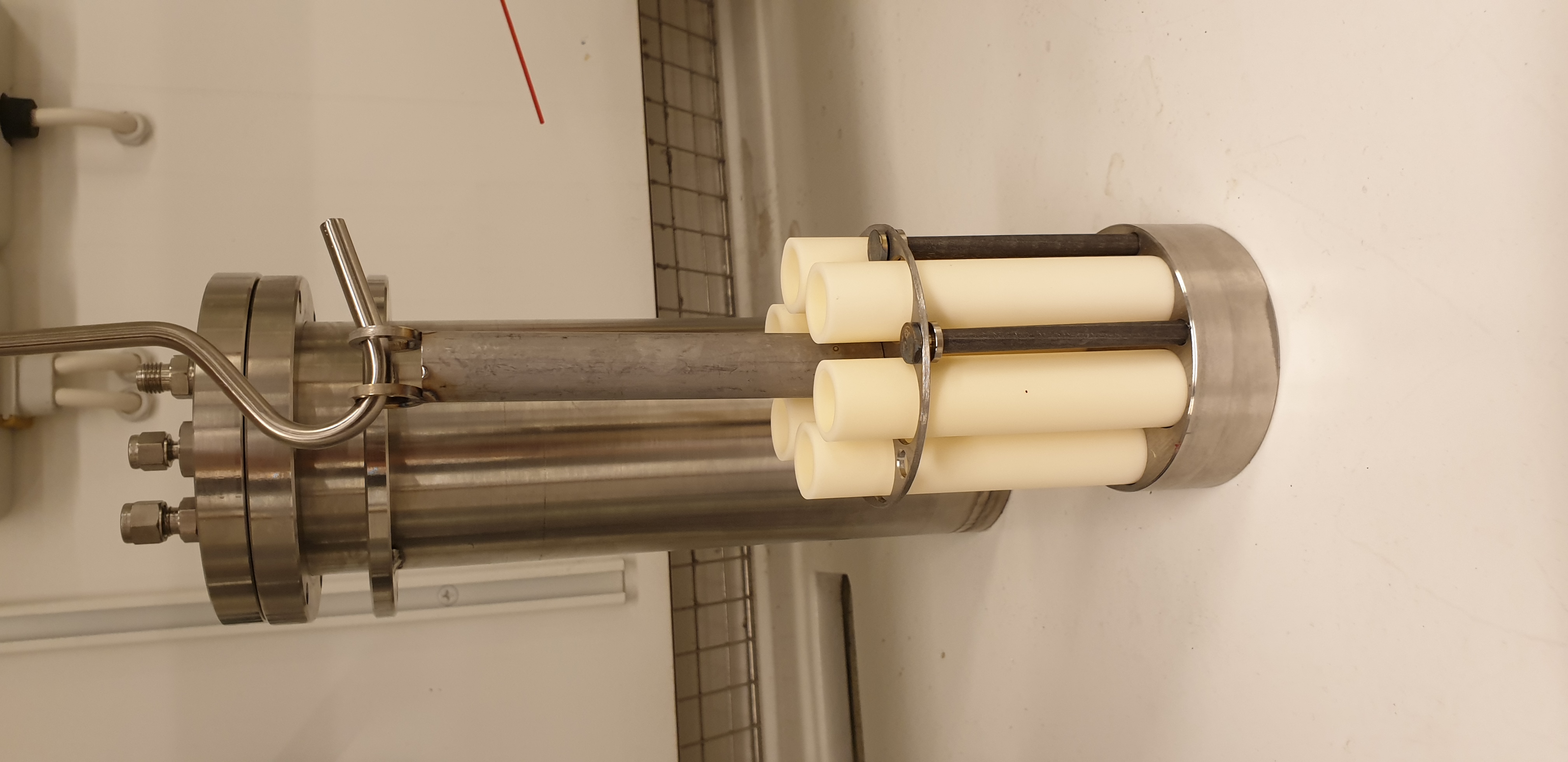

Supplement: Supplementary file 1 [file mmc1.zip › Raw files,figures/longer crucibles.jpg]

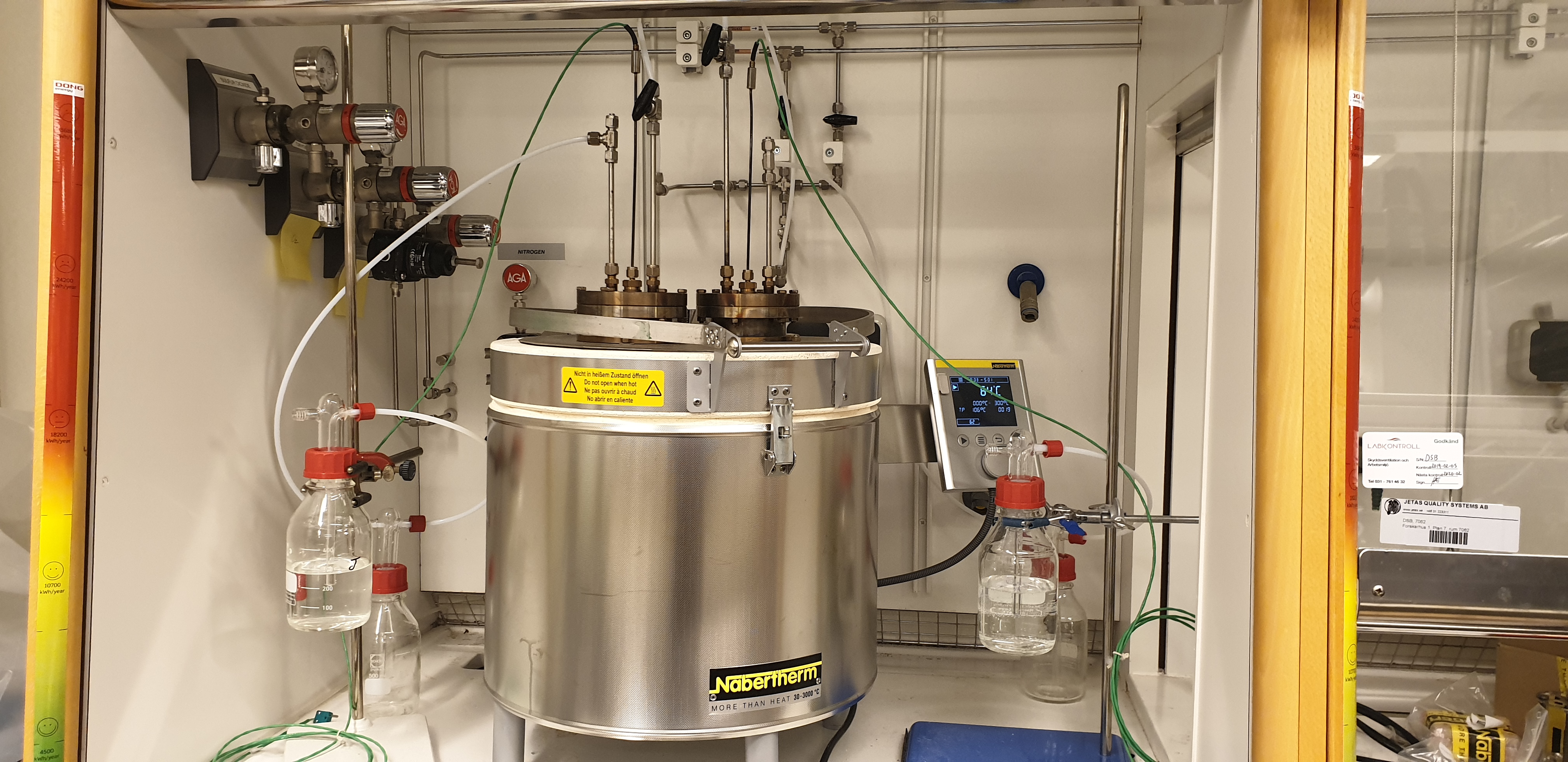

Supplement: Supplementary file 1 [file mmc1.zip › Raw files,figures/New setup.jpg]

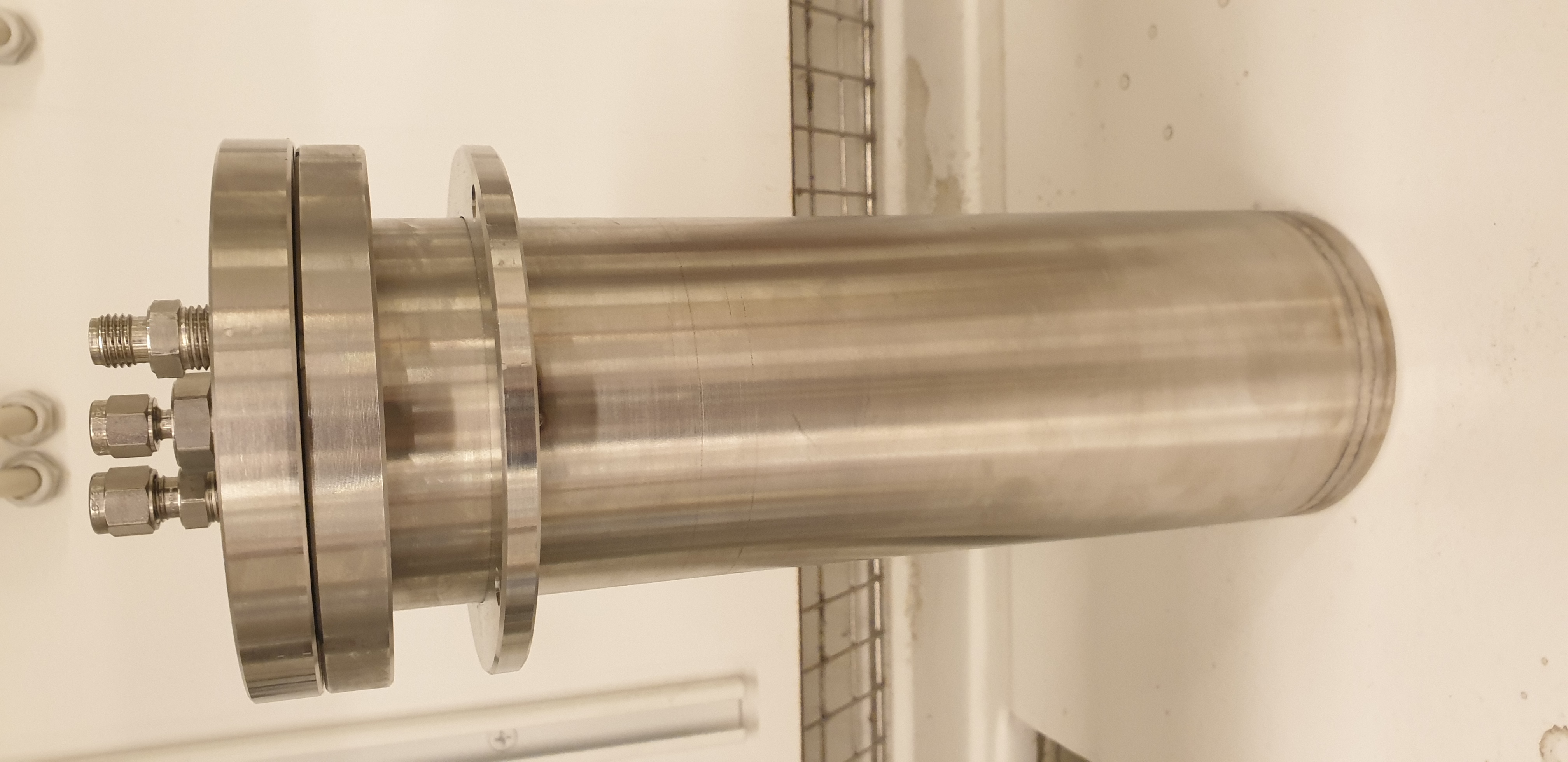

Supplement: Supplementary file 1 [file mmc1.zip › Raw files,figures/vessel.jpg]
