# Supplementary material for: Additional data and experimental setups, for a comparative study of alloys in contact to eutectic melts for thermal storage
Source: Data Brief. 2021 Oct 4;38:107446. doi: 10.1016/j.dib.2021.107446 (PMC8517162; doi:10.1016/j.dib.2021.107446)
Supplement: Supplementary file 2 [file mmc2.zip › Factsage, Tables C1 and C2/Factsage Oct 2020/summary.docx]

2 Al + Li2CO3 + 2 CO2 = 2 LiAlO2 + 3 CO

(1073K) (1073K) (1073K) (1073K) (1073K)

-827188.4

2 Al + Na2CO3 + 2 CO2 = 2 NaAlO2 + 3 CO

(1073K) (1073K) (1073K) (1073K) (1073K)

-786651.4

2 Cr + Na2CO3 + 2 CO2 = 2 NaCrO2 + 3 CO

(1073K) (1073K) (1073K) (1073K,s) (1073K)

-197916.6

69 Cr + 23 Na2CO3 + 40 CO2 = 46 NaCrO2 + Cr23C6 + 57 CO

(1073K) (1073K) (1073K) (1073K,s) (1073K,s) (1073K)

-4852245.1

8 Al + 3 NaNO3 = 3 NaAlO2 + Al2O3 + 3 AlN

(923K) (923K) (923K) (923K) (923K)

-4312939.8

Al + NaNO3 = NaAlO2 + NO

(923K) (923K) (923K) (923K,g)

-675781.3

Cr + NaNO3 = NaCrO2 + NO

(923K) (923K) (923K,s) (923K,g)

-376491.0

23 Fe + 4 NaNO3 = 4 NaFeO2 + Fe3O4 + 4 Fe4N

(923K) (923K) (923K,s) (923K) (923K)

-2024913.6

Fe + NaNO3 = NaFeO2 + NO

(923K) (923K) (923K,s) (923K,g)

-257748.7

8 Cr + 3 NaNO3 = 3 NaCrO2 + Cr2O3 + 3 CrN

(923K) (923K) (923K,s) (923K) (923K)

-2388795.9
